# Exploring and disentangling the production of potentially bioactive phenolic catabolites from dietary (poly)phenols, phenylalanine, tyrosine and catecholamines

**DOI:** 10.1016/j.redox.2024.103068

**Published:** 2024-02-13

**Authors:** Michael N. Clifford, Iziar A. Ludwig, Gema Pereira-Caro, Laila Zeraik, Gina Borges, Tahani M. Almutairi, Sara Dobani, Letizia Bresciani, Pedro Mena, Chris I.R. Gill, Alan Crozier

**Affiliations:** a School of Bioscience and Medicine, Faculty of Health and Medical Sciences, University of Surrey, Guildford, United Kingdom; b Department of Nutrition, Dietetics, and Food, Monash University, Notting Hill, Victoria, Australia; c Center for Nutrition Research, University of Navarra, Pamplona, Spain; d Department of Agroindustry and Food Quality, IFAPA-Alameda Del Obispo, Córdoba, Spain; e Foods for Health Group, Instituto Maimónides de Investigación Biomédica de Córdoba (IMIBIC), Córdoba, Spain; f Human Nutrition Unit, Department of Food and Drug, University of Parma, Parma, Italy; g PolyphenolBio Ltd., Glasgow, United Kingdom; h Department of Chemistry, King Saud University, Riyadh, Saudi Arabia; i Nutrition Innovation Centre for Food and Health, Ulster University, Coleraine, United Kingdom; j Microbiome Research Hub, University of Parma, Parma, Italy; k School of Medicine, Dentistry and Nursing, University of Glasgow, Glasgow, United Kingdom

**Keywords:** Bioactive phenolic catabolites, Dietary (poly)phenolics, Microbiota-mediated catabolism, Endogenous metabolism, Aromatic amino acids, Catecholamines

## Abstract

Following ingestion of fruits, vegetables and derived products, (poly)phenols that are not absorbed in the upper gastrointestinal tract pass to the colon, where they undergo microbiota-mediated ring fission resulting in the production of a diversity of low molecular weight phenolic catabolites, which appear in the circulatory system and are excreted in urine along with their phase II metabolites. There is increasing interest in these catabolites because of their potential bioactivity and their use as biomarkers of (poly)phenol intake. Investigating the fate of dietary (poly)phenolics in the colon has become confounded as a result of the recent realisation that many of the phenolics appearing in biofluids can also be derived from the aromatic amino acids, l-phenylalanine and l-tyrosine, and to a lesser extent catecholamines, in reactions that can be catalysed by both colonic microbiota and endogenous mammalian enzymes. The available evidence, albeit currently rather limited, indicates that substantial amounts of phenolic catabolites originate from phenylalanine and tyrosine, while somewhat smaller quantities are produced from dietary (poly)phenols. This review outlines information on this topic and assesses procedures that can be used to help distinguish between phenolics originating from dietary (poly)phenols, the two aromatic amino acids and catecholamines.

## 1 Introduction

Dietary (poly)phenols, including C_6_–C_3_–C_6_ flavonoids, are found principally as conjugates linked to sugars including glucose and rutinose. Conjugates of C_6_–C_3_ cinnamic acids include quinic and tartaric acid esters. Only relatively small amounts of (poly)phenol conjugates are absorbed without undergoing structural modification. In most instances, after ingestion the attached conjugating moiety is removed in the proximal gastrointestinal (GI) tract by the action of lactase phlorizin hydrolase in the brush border of the small intestine epithelial cells and/or cytosolic β-glucosidase within the epithelial cells. The released aglycones are modified in epithelial/hepatic cells and appear in the bloodstream as phase II sulfate, glucuronide and/or methylated metabolites [1]. Ingested (poly)phenols not absorbed in the upper GI tract pass into the colon where they are subjected to the action of the resident microbiota which catalyse cleavage of the conjugating moiety [[2], [3], [4], [5], [6], [7], [8]]. The released aglycones are subjected to further microbiota-mediated catabolism, including ring fission, which yields a complex mixture of low molecular weight phenolics. These catabolites are absorbed, a portion as phase II metabolites, with what remains being voided in feces [1,9,10].

There is particular interest in colon-derived phenolic catabolites as biomarkers of (poly)phenol intake [[11], [12], [13], [14]]. However, attention is also being focused on their potential involvement in the protective effects of diets rich in fruits and vegetables against the development of non-communicable chronic conditions including coronary heart disease, inflammation, cancer and reduced cognitive function [[15], [16], [17], [18], [19], [20], [22], [23], [25], [26], [27], [28], [29], [30], [31], [32], [33], [34], [35], [36], [37]].

Although largely ignored in recent years, with one notable exception [38], there is compelling evidence that the majority of phenolic catabolites detected in plasma and urine are not unique products of dietary (poly)phenols as they can also originate via a diversity of routes from the aromatic amino acids l-phenylalanine **1** and l-tyrosine **2**, and to a lesser degree catecholamines such as dopamine **3** [39]. This review focusses the origins of low molecular weight phenolics, summarizes studies on the metabolism of, phenylalanine, tyrosine and dopamine, and evaluates protocols that can be used to help distinguish their metabolites from those derived from dietary (poly)phenols. The terminology and synonyms used for phenolic catabolites over the years are confusing and as a consequence the nomenclature proposed by Kay et al. [40] is used throughout this article.

## 2 Evolution of techniques for the analysis of phenolic acids and related compounds

In the 1960s,GC-MS with ∼2.0 mm i. d. rather than capillary GC columns, came into use to for the analysis of phenolics after derivatization to form methyl esters and/or trimethylsilyl ethers. Their phase II metabolites could not be analysed directly as even after derivatization they lacked the volatility necessary to elute from GC columns. In the early 2000's it became possible to analyse phenolics and their phase II conjugates by HPLC-MS without a prerequisite derivatization step. However, at the time the sensitivity of MS detectors was poor and many reference compounds were lacking and as a consequence relatively few phenolics were detected [2,41,42]. Current instrumentation detection limits and selectivity have improved greatly and UHPLC-HR-MS and UHPLC-QQQ-MS instruments are now the analytical platforms of choice for identification and quantification, respectively. As a consequence the number of phenolics that can be monitored has increased markedly in recent years. For instance, Pereira-Caro et al. [43] identified 65 phenolic compounds in urine collected after ingestion of orange juice, while Carregosa et al. [44] reported that in human intervention studies using diets rich in (poly)phenols, 137 low molecular weight phenolics have been detected in plasma.

The large expanding number of phenolics detected in biofluids presents its own analytical problems even with advanced UHPLC-HR-MS and UHPLC-QQQ-MS. Two recent reviews discuss in depth potential pitfalls in the characterization and quantification of (poly)phenol metabolites, and colonic catabolites appearing in biofluids, and how they can be avoided [45,46].

## 3 The fate of dietary (poly)phenols in the colon

**Fig. 1:**
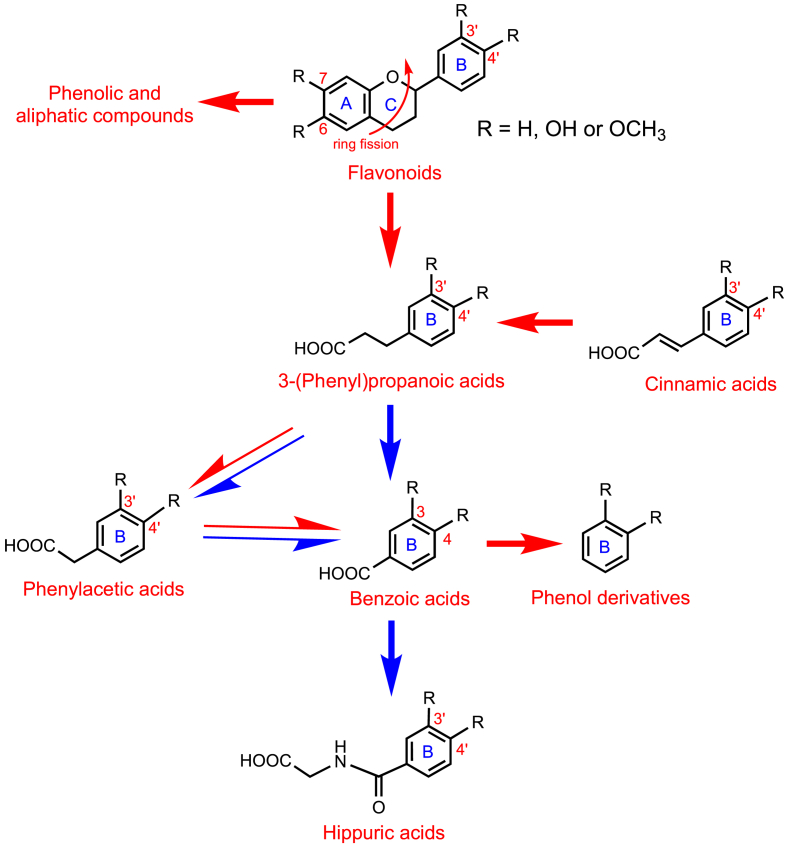
Proposed outline of the pathways for the metabolism of flavonoids and cinnamic acids in the lower gastrointestinal tract after cleavage of the conjugating moiety. Red arrows indicate microbiota-mediated conversions and the blue arrows reactions catalysed by mammalian enzymes. Fine arrows are potential minor routes. Additions to the pathways include the formation of methoxy, glucuronide, and sulfate phase II metabolites. (For interpretation of the references to color in this figure legend, the reader is referred to the Web version of this article.)

Although there are undoubtedly variations on this theme, most notably with flavan-3-ols (see Section 8.1), on entering the colon most C_6_–C_3_–C_6_ flavonoids are subjected to microbiota-mediated O1–C2 and C4–C10 ring fission (Fig. 1) The released A-ring generates phenolic and aliphatic compounds. The cleaved B-ring-forms phenolic acids with a three-carbon side chain, namely phenylpropanoic acids [1]. Cinnamic acids may be absorbed *per se* or after hydrogenation to phenylpropanoic acids [47]. Arguably, most metabolism of the C_6_–C_3_ phenylpropanoic acids involves mammalian enzymes with a β-oxidation catalysing the removal of two carbons from the side chain yielding C_6_–C_1_ benzoic acids. In addition a minor route involving, an α-oxidation or a microbiota-catalysed reaction converts the C_6_–C_3_ phenylpropanoids to C_6_–C_2_ phenylacetic acids which can also be converted to benzoic acids. Benzoic acids, as well as being catabolized yielding phenol derivatives, such as 1,2-dihydroxybenzene (**4**, aka catechol), are metabolized to hippuric acids via a glycination step that is catalysed by hepatic enzymes [1,48] (Fig. 1). Elaborations of the basic pathway include the formation of catabolites with a hydroxylated side chain, such 2*R/S*-hydroxy-3-(4′-hydroxyphenyl)propanoic acid **5**, 3*R/S*-hydroxy-3-(3′-hydroxyphenyl)propanoic acid **6** and 2*R/S*-hydroxy-2-(4′-hydroxyphenyl)acetic acid **7** [39], and the formation of methoxy, glucuronide, and sulfate phase II metabolites [1]. There is considerable inter-person variation in the relative yields of the numerous phenolics and their phase II metabolites [49,50].

**Fig. 2:**
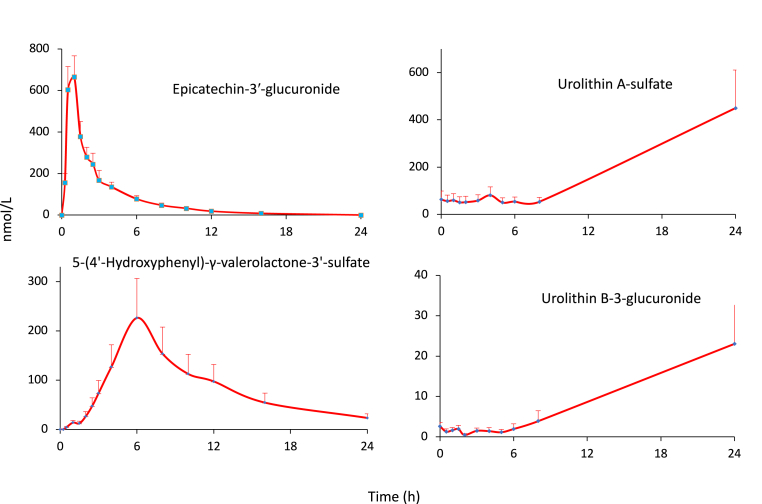
Plasma pharmacokinetic profiles of (−)-epicatechin-3′-glucuronide and 4*R/S*-5-(4′-hydroxyphenyl)-γ-valerolactone-3′-sulfate following the ingestion of [2–^14^C_1_](−)-epicatechin [51,52], and urolithins derived from ellagitannins after the consumption of raspberries [60].

Many phenolic catabolites are common colonic degradation products of a number of different (poly)phenols others, however, have more restricted origins and their presence in biofluids is associated exclusively with the intake of specific (poly)phenols [9]. For example, potentially bioactive C_6_–C_5_ phenyl-γ-valerolactones [26,35,49], such as 4*R/S*-5-(4′-hydroxyphenyl)-γ-valerolactone-3′-sulfate **8**, and phenylvaleric acids including 4*R/S*-hydroxy-5-(3′-hydroxyphenyl)valeric acid-4′-glucuronide **9**, as noted in Section 8.1, are the products of microbiota-catalysed O1–C2 ring fission of (−)-epicatechin **10**, as well as related flavan-3-ol monomers and procyanidins. The C_6_–C_5_ catabolites of (−)-epicatechin have a peak plasma concentration time (*T*_max_) of *ca.* 6–7 h and an apparent elimination half-life (*AT*_*1/2*_) of *ca.* 6 h. They persist in the circulatory system much longer than C_6_–C_3_–C_6_ metabolites, such as (−)-epicatechin-3′-glucuronide **11**, which originate from the small intestine and typically have respective *T*_max_ and *AT*_*1/2*_ times of *ca*. 1 h and 2 h (Fig. 2) [51,52]. Although C_6_–C_5_ valeric acids are also microbiota catabolites of cereal grain alkyl-resorcinols [53] they differ from the flavan-3-ol phenylvaleric acids as they lack a side chain hydroxyl group.

Another example is the urolithins, catabolites of ellagitannins such as sanguiin H-6 **12**, which is found in raspberries, and punicalagin **13** and punicalin **14** which occur in pomegranates [[54], [55], [56], [57]]. Ellagitannins are absorbed poorly, if at all, in the upper GI tract and on reaching the colon undergo microbiota-mediated conversion to urolithins, but first they are hydrolysed by tannin-hydrolase and the released hexa-hydroxydiphenic acid undergoes spontaneous lactonization to form ellagic acid **15** [58]. Lactone-ring cleavage and decarboxylation of ellagic acid yields a pentahydroxy-urolithin, 3,4,8,9,10-pentahydroxy-urolithin (**16**, aka urolithin M5). Dehydroxylation reactions then occur generating a series of tetrahydroxy-trihydroxy-, dihydroxy- and finally a mono-hydroxy-urolithin [58]. The urolithins form phase II conjugates and urinary excretion starts *ca.* 16 h post-ellagitannin ingestion and can continue for a further 4 days [57,59]. They are also long-lived in the circulatory system compared to other phenolic catabolites, including phenyl-γ-valerolactones, first appearing in the circulatory system *ca*. 8 h after ellagitannin intake (Fig. 2) [60] and attaining peak plasma concentrations (*C*_max_) of up to 5 μmol/L [58,59].

There is a large inter-individual variability in the levels of urolithins, and this has been associated with varying colonic microbiota compositions. The variability is not only quantitative with low vs. high producers [58], but also qualitative with more subjects excreting 3,8-dihydroxy- than 3-hydroxy-urolithin derivatives [57,58], leading to the concept of metabolic phenotypes [57,61,62]. There is evidence that obese subjects producing 3-hydroxyurolithin **17** (aka urolithin B) and 3,9-dihydroxy-urolithin **18** (aka isourolithin A) are at increased risk of cardiovascular disease while a 3,8-dihydroxy-urolithin (**19**, aka urolithin A) phenotype provides protection [63]. A comprehensive evaluation of the metabolism and bioactivity of urolithins and the involvement of the gut microbiota has been published by Garcia-Villalba et al. [64].

Isoflavones and lignans can be added to the aforementioned flavan-3-ols and procyanidins and ellagitannins as classes of (poly)phenols that give rise to unique colon-derived catabolites. Isoflavones are converted to *S-*equol **20**, desmethylangolensins, such as *O*-desmethylangolensin **21**, and 2-(phenyl)propanoic acid **22** [65], while lignans yield enterodiol **23** and enterolactone **24** [66].

## 4 Feeding protocols for the investigation of colonic catabolites of dietary (poly)phenols

A frequently used protocol in human clinical bioavailability studies with test meals or beverages has involved collecting urine over a 12 h overnight fasting period after adherence to a low (poly)phenol diet for at least 36 h. Participants then ingest the test food/beverage after which plasma and urine are collected at intervals over a 24 h or 48 h period during which time they continue the restricted low (poly)phenol diet. The collected fluids are analysed by UHPLC-HR-MS and/or UHPLC-QQQ-MS.

Cumulative washout in urine indicates the percentage recovery of ingested (poly)phenols as metabolites and catabolites. In view of the presence of background low molecular weight phenolics, the quantity of phenolics in 12 h pre-feed baseline urine is used on a per-hour basis to subtract from the 0–24/0-48 h post-feed levels, thus minimising the risk of over-estimating phenolics produced from the ingested supplement.

Typical data obtained with this approach in a bioavailability study by Ludwig and co-workers [60], in which raspberries containing of 292 μmol of anthocyanins, principally cyanidin-3-*O*-sophoroside **25**, were ingested by 9 subjects, are presented in Table 1. Excretion of anthocyanins was a mere 0.007% of intake, however, significant increases in 14 phenolic acids were noted with a substantial amount of hippuric acid **26** dominating both baseline phenolics and those excreted post-raspberry consumption. There were marked person to person variations with both pre- and post-supplement excretion of hippuric acid with amounts excreted by some subjects being substantially higher than the 292 μmol of ingested anthocyanins. The large quantity of hippuric acid in baseline urine demonstrates that it originates from sources other than the ingested anthocyanins. Excretion of phenolics, without taking hippuric acid into account, was equivalent to 15% of the anthocyanin intake (Table 1).

**Table 1:** Increased urinary excretion of phenolic acids 0–48 h after acute intake of 300 g of blended raspberries containing 292 μmol of anthocyanins (99.2% cyanidin-based)[60] a.

Phenolic acids	Baseline^b^	Total (0–48 h)^c^
*Cinnamic acids*		
4′-Hydroxycinnamic acid-3′-sulfate	–	1.1 ± 0.1
4′-Hydroxy-3′-methoxycinnamic acid	1.8 ± 0.3	5.9 ± 2.1
3′-Methoxycinnamic acid-4′-sulfate	1.6 ± 0.2	6.5 ± 2.6
3′-Methoxycinnamic acid-4′-glucuronide	0.7 ± 0.1	1.8 ± 0.5
4′-Methoxycinnamic acid-3′-sulfate	–	0.5 ± 0.2
4′-Methoxycinnamic acid-3′-glucuronide	0.3 ± 0.1	1.0 ± 0.4
*Total cinnamic acids*	*4.4 ± 0.4*	*16.8 ± 3.6*
*Phenylpropanoic acids*		
3-(4′-Hydroxypheny)propanoic acid-3′-sulfate	–	1.1 ± 0.3
*Total phenylpropanoids*	*–*	*1.1 ± 0.3*
*Phenylacetic acids*		
3′,4′-Dihydroxyphenylacetic acid	0.4 ± 0.2	2.9 ± 1.3
4′-Hydroxy-3′-methoxyphenylacetic acid	–	0.2 ± 0.2
*Total phenylacetic acids*	*0.4 ± 0.2*	*3.1 ± 1.7*
*Benzoic acids*		
3-Hydroxybenzoic acid-4-sulfate	0.1 ± 0.0	0.2 ± 0.2
4-Hydroxybenzoic acid-3-sulfate	0.1 ± 0.0	0.3 ± 0.1
4-Hydroxybenzoic acid	–	6.4 ± 4.8
*Total benzoic acids*	*0.2 ± 0.0*	*6.9 ± 5.0*
*Hippuric acids*		
4′-Hydroxyhippuric acid	7.0 ± 2.1	16.1 ± 1.9
Hippuric acid	294 ± 47	239 ± 55
*Total phenolics excluding hippuric acid*	*12.0 ± 2.3*	*44.0 ± 8.1 (15%)*^d^

a Data expressed in μmol as mean values ± SE (n = 9).

b Subjects were on a 0–36 h a low (poly)phenol diet, after which they fasted for 12 h and baseline urine excreted during this period was collected prior to the ingestion of raspberries.

c Phenolic acids collected 0–48 h following raspberry intake after subtraction on an excretion per hour basis, of baseline excretion values. All listed phenolics exhibited significantly higher excretion above baseline values (p < 0.05).

d The amount excreted as a percentage of the 292 μmol of ingested anthocyanins.

**Fig. 3:**
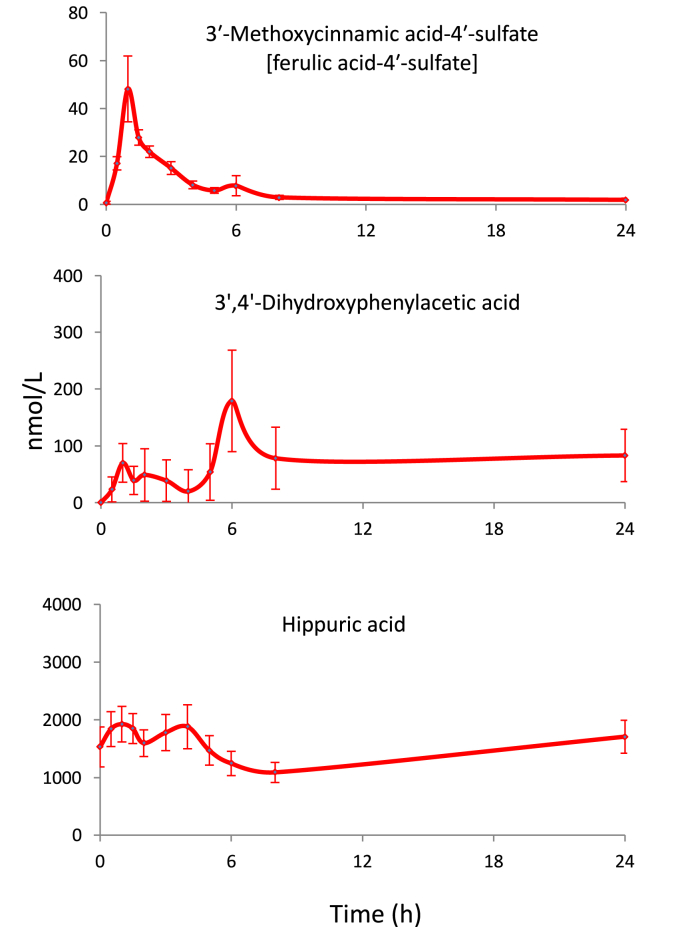
Plasma pharmacokinetic profiles of 3′-methoxycinnamic acid-4′-sulfate, 3′,4′-dihydroxyphenylacetic acid and hippuric acid following the ingestion of 300 g of raspberry purée [60].

The time of appearance of phenolics in the circulatory system indicates whether absorption has occurred in the upper or lower GI tract, or both, as illustrated by the Ludwig et al. raspberry study [60]. Fig. 3 illustrates the plasma profiles of 3′-methoxycinnamic acid-4′-sulfate **27**, 3′,4′-dihydroxyphenylacetic acid **28** and hippuric acid **26** obtained after the ingestion of raspberries. The cinnamic acid has a *T*_max_ of 1 h characteristic of metabolism and absorption in the small intestine while the longer 6 h *T*_max_ of 3′4′-dihydroxyphenylacetic acid indicates it is a product of catabolism in the lower GI tract. In keeping with the urine data, the major plasma phenolic was hippuric acid with a plasma concentration of *ca*. 2000 nmol/L which changed little following raspberry consumption. This along with its high baseline level of urinary excretion indicates that hippuric acid originates principally from compounds other than the ingested anthocyanins and estimating what would appear to be the relatively small amounts derived from the raspberry anthocyanins is not feasible. The most obvious source would be benzoic acid but it was not detected in the raspberries. Similarly high levels of hippuric acid have been obtained in other bioavailability studies [39,[67], [68], [69], [70]].

Arguably, the best strategy to minimise the risk of over-inflating the yield of metabolites associated with the test meal/beverage is to extend the washout period until the excretion of these metabolites has become asymptotic. Washout of metabolites sequestered in tissues and/or bound to serum albumin has rarely been studied explicitly but metabolites from this source may still be detected in urine after two days on a low (poly)phenol diet, and as a consequence require a longer washout period. This is almost certainly the case with metabolites that originate from ellagitannins, and possibly proanthocyanidins, and thearubigins, and even some simple flavonoids, which are capable of binding strongly to the gut mucosa and thus expanding the period over which absorption and excretion occurs [71]. Washout periods may also be extended by matrix effects such as the presence of fibre with the ingested supplement [72]. Ideally they should be longer than 48 h. However, there are practical limitations as to how long volunteers are willing to follow a low (poly)phenol diet, although arguably less than two days is likely to compromise the results by reducing the yield, and possibly distorting the metabolite profile. Moreover, it precludes access to washout data of value in exploring, among other things, tissue accumulation which is otherwise little studied.

Fecal samples have been analysed only rarely in bioavailability investigations [51,73,74] despite the information obtained being very relevant to determining the fraction of phenolic catabolites that is not absorbed and are potentially bioactive in the colon. However, anaerobic *ex vivo* incubation of (poly)phenols and related compounds with gut bacteria and fecal material have been widely used and has provided key information on microbiota-mediated catabolism [34,38,42,68,70,[75], [76], [77], [78], [79], [80], [81]], but not the accompanying *in vivo* phase II metabolism conversions that are catalysed by mammalian enzymes [43,47,51,67,69,82]. *Ex vivo* incubations with specific substrates, especially those labelled with ^13^C or ^2^H, can provide valuable information. With unlabelled substrates, the use of phenylalanine- and tyrosine-free media, and blank incubations, are able to preclude inputs from protein degradation products that can complicate matters as discussed in Section 6.

A further approach to define the role of the gut microbiota is to perform feeding studies with healthy volunteers and healthy ileostomists who have had their colon removed surgically. The plasma, urine, and feces of healthy volunteers with an intact colon include metabolites originating from the colonic microbiota, but these are not present in samples provided by the ileostomists because their colon has been removed [[2], [3], [4], [5], [6], [7], [8],^82^,^83^]. Some metabolites such as the free cinnamic acids may be absorbed in the stomach or the small intestine by both groups, while the portion, often substantial, released from unabsorbed conjugates by the colonic microbiota will not be produced by the ileostomists [5]. Analysis of their ileal effluent will reveal these untransformed substrates plus any metabolites excreted in bile and/or effluxed from the upper GI tract enterocytes. These substrates and metabolites cannot otherwise be discriminated and quantified because they are further transformed by the colonic microbiota and may be absorbed and recycled in volunteers with an intact colon.

## 5 Phenolic compounds derived from amino acids and catecholamines

Phenylalanine **1** is an essential amino acid as it is not synthesized *de novo* in animals and must be provided by the diet. It occurs in many animal and plant-derived foods including beef, pork, lamb, venison, poultry, tuna, cottage cheese, milk, lentils, soy beans, peanuts and fruits [84]. In the body it can undergo hepatic 4′-hydroxylation to form tyrosine **2.** Both aromatic amino acids are incorporated into proteins, and released during protein breakdown when there is no entry of the amino acids into the body from dietary sources [85].

Some of the background phenolics found in plasma and urine, especially hippuric acid as noted in Section 5, are not derived exclusively from dietary (poly)phenols. There are reports that they are also catabolites of the aromatic amino acids phenylalanine, tyrosine, and, to a lesser degree, as they occur in much smaller amounts, catecholamines including dopamine **3**. Compelling evidence on the involvement of these compounds can be found in papers from the 1960s and 1970s which until recently have escaped notice [39].

In 1961 Grümer reported that [^14^C]phenylalanine **1** administered intravenously to phenylketonuric subjects, where conversion to tyrosine is blocked, was converted to radiolabelled hippuric acid **26** which was detected in urine collected for a 6 h period with a 4.7% yield [86]. This indicates that the conversion of phenylalanine to hippuric acid was probably hepatic in origin and had by-passed the gut microbiota.

**Fig. 4:**
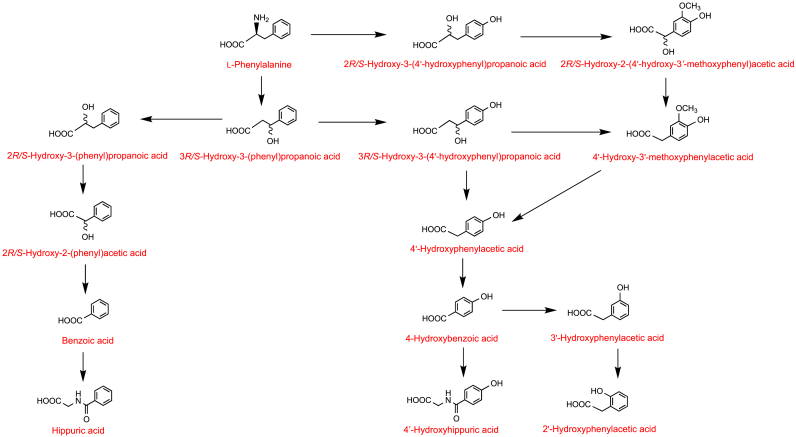
Potential pathways for the metabolism of deuterated phenylalanine following oral intake. The arrows usually represent more than one metabolic step, sometimes many more. Based on data of Curtius et al. [87,88].

**Fig. 5:**
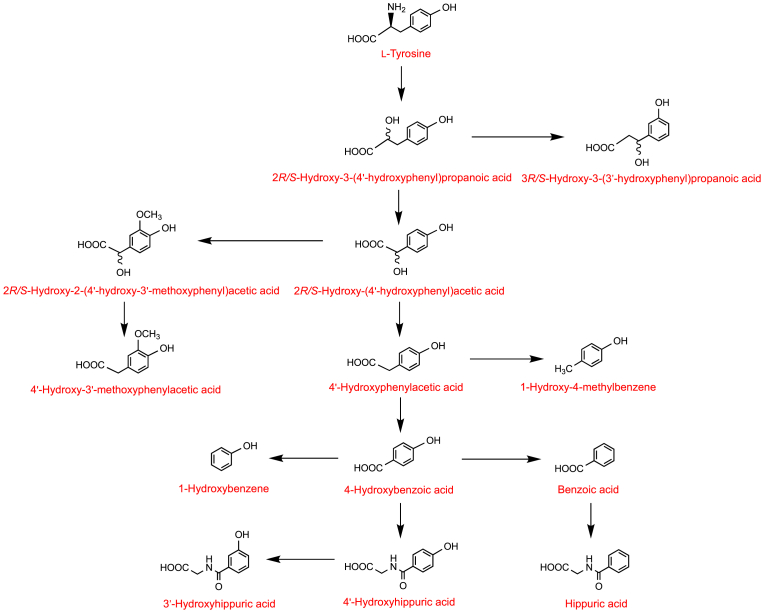
Potential pathways for the metabolism of deuterated tyrosine following oral intake. The arrows usually represent more than one metabolic step, sometimes many more. Based on data of Curtius et al. [87,88].

In 1972 and 1975, Curtius and colleagues reported on the metabolism of deuterated phenylalanine and tyrosine ingested orally by healthy subjects, participants with hyperphenylalaninemia and phenylketonuria, and mentally retarded patients [87,88]. These studies used GC-MS to identify labelled metabolites in urine which allowed metabolic pathways to be inferred but provided little quantitative information, and none on phase-II conjugates. Potential pathways of the metabolism of phenylalanine and tyrosine are illustrated, respectively, in Fig. 4, Fig. 5. Hippuric acid **26** and 4′-hydroxyphenylacetic acid **29** were the main metabolites of [^2^H_2_]phenylalanine which was also converted to benzoic acid **30** and 4′-hydroxyhippuric acid **31.**

When subjects were given the antibiotic neomycin for 3 days pre-feed to inhibit the gut microbiota, ingested ^2^H_2_-tyrosine was no longer converted to benzoic acid and hippuric acid although deuterated 4-hydroxybenzoic acid **32** and 4′-hydroxyhippuric acid **31** were still detected in urine [88]. This finding suggests that in the upper GI tract a portion of the ingested [^2^H_2_]tyrosine is absorbed into the circulatory system and undergoes hepatic side chain shortening, resulting in the formation of 4-hydroxybenzoic acid and conversion to 4′-hydroxyhippuric acid. Tyrosine remaining in the GI tract passes to the colon where the microbiota catalyse aliphatic side chain shortening and 4′-dehydroxylation with the resultant benzoic acid **30** being absorbed and converted to hippuric acid **26**. As a consequence, pre-feed treatment with neomycin inhibited the gut microbiota and therefore blocked hippuric acid production but not that of 4′-hydroxyhippuric acid [86]

**Fig. 6:**
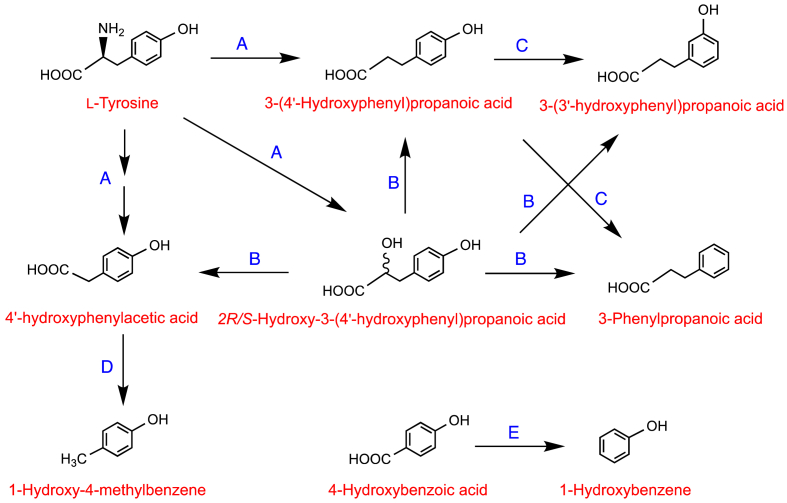
Proposed catabolism of deuterated tyrosine and related phenolics following fecal incubations. Routes from (**A**) [^2^H_2_]tyrosine, (**B**) [^2^H_2_]2-hydroxy-3-(4′-hydroxyphenyl)propanoic acid, (**C**) [^2^H_2_]3-(4′-hydroxyphenyl)propanoic acid, (**D**) [^2^H_4_]4′-hydroxyphenylacetic acid and (**E**) [^2^H_2_]4-hydroxybenzoic acid. The arrows usually represent more than one metabolic step, sometimes many more. Based on data of Curtius et al. [89].

Deuterated tyrosine **2**, 2*R/S*-hydroxy-3-(4′-hydroxyphenyl)propanoic acid **5**, 3-(4′-hydroxyphenyl)propanoic acid **33**, 4′-hydroxyphenylacetic acid **29** and 4-hydroxybenzoic acid **32** were all used as substrates in anaerobic incubations with fecal material [89]. The resultant conversions are presented in the pathways illustrated in Fig. 6. It is of note in Fig. 6 that in some instances 4′-hydroxylated C_6_–C_3_ phenolics were converted by the microbiota to 3′-hydroxylated products, such as the transformation of 3-(4′-hydroxyphenyl)propanoic acid **33** to 3-(3′-hydroxyphenyl)propanoic acid **34**. This is based on an NIH-shift of the aliphatic side chain, a conversion in which certain anaerobic species move the three carbon side chain of tyrosine and/or the associated 4′-metabolites around the ring such that the 4′-hydroxyl becomes a 3′-hydroxyl [90].

The third aromatic amino acid tryptophan **35**, is a precursor for serotonin **36** synthesis and, in normal conditions, its degradation in mammals involves hepatic conversions to picolinic acid **37**, quinolinic acid **38**, and a number of indole derivatives, while a branch in the brain proceeds to kynurenine **39**. One of the products of kynurenine is 1,2-dihydroxybenzene **4** which is degraded to water and carbon dioxide [91]. This may not be relevant to circulating levels of 1,2-dihydroxybenzene, and in any case the amount of the benzene derivatives produced via this route are likely to be extremely small compared to that originating from phenylalanine, tyrosine and dietary (poly)phenols.

**Fig. 7:**
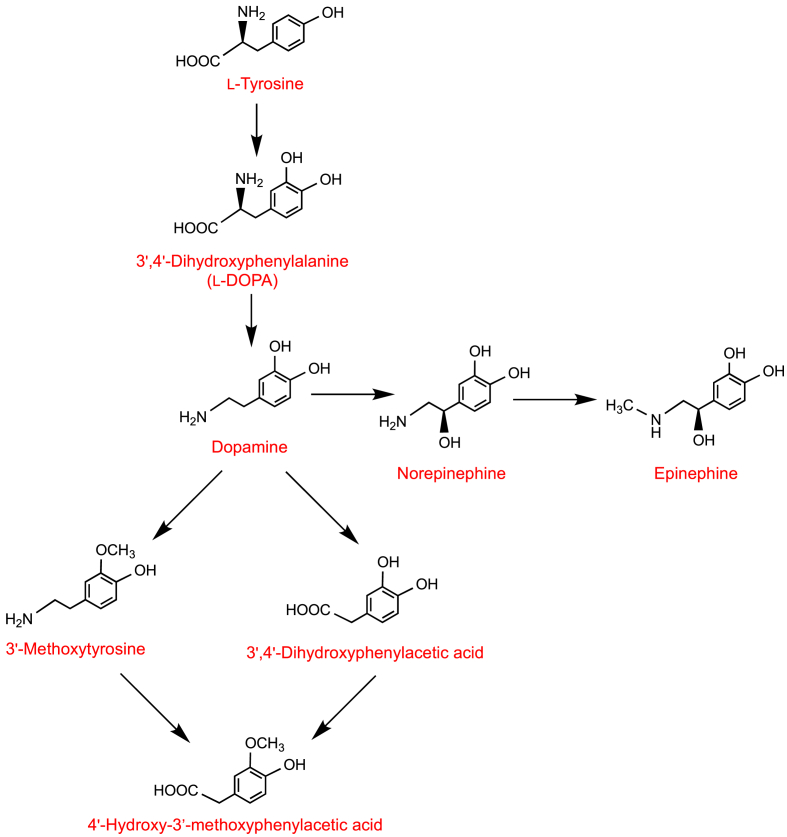
Synthesis and catabolism of dopamine and related catecholamines. After Olguín et al. [94].

Catecholamine synthesis *in vivo* begins with dietary phenylalanine and/or tyrosine being metabolized to 3′,4′,dihydroxyphenylalanine (**40,** l-DOPA) that is decarboxylated yielding dopamine **3** [[92], [93], [94]]. In turn, dopamine is converted to norepinephrine and epinephrine (aka adrenaline) (see Fig. 7) in adrenomedullary chromaffin cells. Norepinephrine is also produced by sympathetic nerve endings. Only a small fraction of catecholamines is released from storage vesicles of sympathetic nerves and enters the circulation. Dietary sources of dopamine, and other catecholamines, undergo sulfation in the GI tract before entering the bloodstream and, as a consequence, almost all circulating forms are sulfate derivatives. Extraneural metabolism of dopamine by catechol-*O*-methyltransferase and monoamine oxidase leads to the formation of 3′-methoxytryptamine, 3′,4′-dihydroxyphenylacetic acid and 4′-hydroxy-3′-methoxyphenylacetic acid as illustrated in Fig. 7 [94]. 3′,4′-Dihydroxyphenylacetic acid **28** is a biomarker of catecholamine-secreting tumors. However, false positive results can occur [95,96] as the dihydroxyphenylacetic acid is also a colonic catabolite of the dietary flavonol quercetin **41** which as glycoside conjugates occurs widely in fruits and vegetables, and is found in onions in especially high concentrations [56]. However, the contribution of dopamine to the phenolic acid catabolite pool, like that of tryptophan, is likely to be very small compared to that of phenylalanine and tyrosine.

## 6 Phenolics excreted in urine by subjects on a low (poly)phenol diet

An investigation involving participants with a full GI tract and ileostomists has provided insights into the origins of at least some of the “background” phenolics in feeding studies with test meals or beverages. Urine collected from the two groups of subjects over a 12 h fasting period, after prior adherence to a low (poly)phenol diet for 36 h, was subjected to detailed analysis by HPLC-HR-MS [39]. Over 70 phenolics were detected and quantified. Many were present in sub-μmol amounts excreted in statistically similar amounts by both groups. Total excretion of the different categories of phenolics are summarised in Table 2 along with information on key individual phenolics which were excreted in μmol amounts by subjects with a full GI tract and, in some but not all instances, lower quantities by ileostomists.

**Table 2:** Urinary excretion of selected phenolics by participants with (n = 8) and without a colon (n = 10), who were on a low (poly)phenol diet for 0–36 h, after which they fasted for 12 h. Urine collected in the 36–48 h period of the study was analysed by UHPLC-HR-MS [39] a.

Phenolics	With colon	Without colon	ANOVA p value
*Cinnamic acids (13)*			
*Total cinnamic acids*	*3.8 ± 0.4*	*4.8 ± 0.4*	ns
*Phenylpropanoic acids (14)*			
3-(3′-Methoxyphenyl)propanoic acid-4′-sulfate	2.4 ± 0.2	0.33 ± 0.03	*
3-(Phenyl)propanoic acid-4′-sulfate	1.2 ± 0.2	0.04 ± 0.00	*
*Total phenylpropanoic acids*	*23.5 ± 2.2*	*21.5 ± 3.2*	ns
*Phenylhydracrylic acids (3)*			
3*R/S*-Hydroxy-3-(3′-hydroxy-4′-methoxyphenyl)propanoic acid	4.9 ± 0.3	4.8 ± 0.3	ns
3*R/S*-Hydroxy-3-(4′-hydroxy-3′-methoxyphenyl)propanoic acid	3.1 ± 0.3	0.6 ± 0.05	*
3*R/S*-Hydroxy-3-(3′-hydroxyphenyl)propanoic acid	13.4 ± 1.1	6.1 ± 0.3	*
*Total phenylhydracrylic acids*	*21.4 ± 1.7*	*11.5 ± 0.7*	*
*Phenylacetic acids (10)*			
3′4′-Dihydroxyphenylacetic acid	10.5 ± 0.9	12.6 ± 0.9	ns
3′-Methoxyphenylacetic acid-4′-sulfate	1.3 ± 0.08	1.2 ± 0.1	ns
4′-Methoxyphenylacetic acid-3′-sulfate	2.6 ± 0.4	2.8 ± 0.5	ns
*Total phenylacetic acids*	*19.0 ± 1.8*	*23.6 ± 1.9*	ns
*Mandelic acids (2)*			
2*R/S*-Hydroxy-2-(4′-hydroxyphenyl)acetic acid	13.4 ± 1.2	10.8 ± 0.4	ns
2*R/S*-Hydroxy-2-(4′-Hydroxy-3′-methoxyphenyl)acetic acid	19.3 ± 1.3	17.8 ± 1.0	ns
*Total mandelic acids*	*32.7 ± 2.5*	*28.6 ± 1.4*	ns
*Benzoic acids and aldehydes (16)*			
3,4-Dihydroxybenzoic acid	4.3 ± 0.6	0.65 ± 0.06	*
Benzoic acid-sulfate-1	6.7 ± 0.8	0.80 ± 0.06	*
*Total benzoic acids and aldehydes*	*25.4 ± 3.3*	*18.7 ± 1.7*	ns
*Benzene catabolites (15)*			
1-Hydroxybenzene-2-sulfate	14.9 ± 1.1	5.1 ± 0.3	*
Methoxybenzene-sulfate-2	22.2 ± 2.1	1.5 ± 0.1	*
Hydroxy-methoxybenzene-sulfate-1	15.3 ± 0.8	4.5 ± 0.7	*
Hydroxy-methoxybenzene-sulfate −2	4.2 ± 0.3	1.4 ± 0.2	*
*Total hydroxybenzenes*	*80.6 ± 7.5*	*33.2 ± 3.3*	*
*Hippuric acids (4)*			
Hippuric acid	366 ± 28	238 ± 13	ns
3′-Hydroxyhippuric acid	17.3 ± 2.3	0.90 ± 0.04	*
4′-Hydroxyhippuric acid	19.9 ± 1.4	12.3 ± 0.5	ns
*Total hippuric acids*	*403 ± 32*	*252 ± 14*	ns
***Total phenolics***	***609 ± 51***	***394 ± 27***	*

Asterisks denote a statistically significant difference among two groups of subjects.

*p value < 0.05; ns – not statistically significant; n.d. not detected.

Figures in italicized parentheses are the total number of phenolics detected in each category including those excreted in sub-μmol amounts that were not statistically different. The total quantities of phenolics excreted in each phenolic category are presented in italics.

a Data expressed as mean values in μmol ± SE.

Hippuric acid **26** was by far the major aromatic detected in the study with 366 ± 28 μmol for participants with a colon and 238 ± 13 μmol excreted by the ileostomists. The two levels of excretion are not statistically different because of large differences in the amounts excreted by individual subjects. Hippuric acid dominated accounting on average for 60% of the total excreted phenolics for both volunteer categories. This implies substantial production of hippuric acid, from surplus phenylalanine and/or tyrosine, and to a lesser degree because of their lower levels, from catecholamines, via endogenous routes independent of the colonic microbiota. Potential intermediates in these conversions, such as benzoic acid **30**, were present in much lower levels, presumably as a result of being rapidly turned over and converted to hippuric acid. It is of note, however, that there were similar levels of excretion by volunteers with an intact colon and ileostomists of 2*R/S*-hydroxy-2-(4′-hydroxyphenyl)acetic acid **7** (13.4 μmol vs 10.8 μmol), 2*R/S*-hydroxy-2-(4′-hydroxy-3′-methoxyphenyl)acetic acid **42** (19.3 μmol vs 17.8 μmol), and 3′,4′-dihydroxyphenylacetic acid **28** (10.5 μmol vs 12.6 μmol) [39], all known metabolites of tyrosine **2** [88,89].

Eleven phenolics were excreted in significantly larger amounts by subjects with a colon than by ileostomists (Table 2). Among the compounds in this category were two phenylhydracrylic acids, two benzoic acid derivatives, four sulfo-benzene conjugates and 3′-hydroxyhippuric acid **43**. The sulfo-benzene metabolites were the largest group of compounds in this category. These phenolics are likely to be produced principally from tyrosine and phenylalanine in the low (poly)phenol diet which will also contain proteins that are broken down in the small intestine by the action of chymotrypsin, trypsin and releasing further quantities of the two aromatic amino acids.

It is evident that the metabolic pathways associated with phenylalanine, tyrosine and catecholamines are complex and overlap with the pathways originating from most dietary (poly)phenols and to disentangle them is often difficult/impossible. It is, for instance, not possible, absolutely, to determine the origin of many of the “background” phenolics in Table 2. For example, as noted above, phenolics of tyrosine/catecholamine metabolism, such as 3′,4′-dihydroxyphenylacetic acid **28** and 4′-hydroxy-3′-methoxyphenylacetic acid (**44**, aka homovanillic acid) [96] are also colonic catabolites of quercetin **41** [2].

Yet further complexities arise with the microbiota-mediated aliphatic side chain shift of certain 3′- and 4′-hydroxy C_6_–C_3_ metabolites [90]. This overlaps with 3′-hydroxyphenyl catabolites produced by the gut microbiota 4′-dehydroxylation of 3′,4′-dihydroxy C_6_–C_3_ substrates which frequently dominate phenolics derived from dietary (poly)phenols. Clearly, excluding these shared metabolites will under-estimate the true yield from the test meal/beverage (poly)phenols, but including them will lead to an over-estimate of the yield. However, if such a metabolite is bioactive and has some potentially beneficial effect does it matter whether it is derived from amino acid or the catecholamines rather than dietary (poly)phenols?

In most instances it is difficult/impossible to determine to what extent bioactive phenolics are derived from dietary (poly)phenols or alternative sources. However, plasma pharmacokinetic profiles can be of assistance. For instance, the profile of hippuric acid **26** in Fig. 3 is in keeping with most of the glycinated benzoic acid being derived from phenylalanine and tyrosine released from breakdown of endogenous proteins. Information on plasma profiles obtained with ileostomists could also be of value. In addition, more clarity will become apparent when, as discussed in Section 4, the ingested dietary supplement contains (poly)phenols that yield metabolites that have never been reported in studies of amino acid or catecholamine metabolism. These are of particular interest because any physiological effect associated with them can be more readily traced back to the test meal/beverage.

## 7 (Poly)phenol bioavailability studies using isotopically-labelled substrates

The use of isotopically-labelled (poly)phenol substrates in bioavailability feeding studies is the gold standard to discriminate between phenolics derived from (poly)phenolics in the colon and unlabelled products in biofluids originating from surplus amino acids, catecholamines and other sources. However, this approach has rarely been used. There are only two human (poly)phenol feeding studies reported between 2012 and 2016 that have employed such methodology, one with ^14^C- labelled (−)-epicatechin **10** [51,52], and the other with ^13^C-labelled cyanidin-3-*O*-glucoside **45** [74,97]. In part, this is likely to be because of the difficulties in obtaining appropriately labelled substrates, that have been approved for human consumption. Obtaining ethical permission for investigations with radiolabeled substrates is not straight forward and when it is obtained rigorous conditions are applied under which the feeding studies can be carried out, and this is reflected in the incumbent associated costs. For instance, after the intake of ^14^C-labelled (−)-epicatechin individual participants were only allowed to be discharged from the research facility after 6–9 days, when radioactivity excreted in urine and voided feces over 24 h was <1% of the administered dose [51].

### 7.1 [2–^14^C_1_](−)-Epicatechin

A feeding study reported by Ottaviani et al. [51] utilized radiolabelled (−)-epicatechin. Eight male subjects ingested 300 μCi (270 μmol) of [2–^14^C_1_](−)-epicatechin ([2–^14^C]EC) after which radioactivity in blood, urine and feces, collected at regular intervals over a period of up to 8 days, were initially monitored using liquid scintillation counting. This revealed an almost total recovery of radioactivity in 0–48 h urine (82 ± 5%) and feces (12 ± 3%) indicating minimal long term tissue deposition of the compounds derived from the ingested flavan-3-ol monomer. Urine collected from participants post-48 h contained negligible amounts of radioactivity demonstrating almost complete 0–48 h washout of the [2–^14^C]EC and its derived products. Radioactivity in feces was voided in a more irregular manner and over a longer 5-day period without a concomitant appearance of radioactivity in plasma or urine, arguably as a consequence of the ^14^C-labelled catabolites being bound to fecal material in the colon. This clearly shows that washout metabolites from tissues via urine did not extend beyond 48 h. However, it cannot be assumed that also applies when (poly)phenols are ingested along with more complex food matrices.

HPLC-MS-MS detected 12 glucuronide, sulfate and methylated metabolites, referred to as structurally related (−)-epicatechin metabolites (SREMs), in plasma [51,52]. After attaining an overall *C*_max_ of 1223 nmol/L with a *T*_max_ of *ca.* 1.0 h after intake of [2–^14^C]EC), the SREMs declined rapidly with an apparent elimination half-life (*AT*_*1/2*_) of 1.9 h and, in almost all instances, had disappeared from the circulatory system within 8 h. The *T*_max_ is indicative of absorption in the upper GI tract (see Fig. 2). A series of microbiota-derived C_6_–C_5_ ring fission catabolites (5C-RFCs), with a summed *C*_max_ of 588 nmol/L appeared in plasma with *T*_max_ of *ca*. 6 h. They were present in the circulatory systems for longer than the SREMs having an *AT*_*1/2*_ of 5.7 h. The main 5C-RFC was 4*R/S*-5-(4′-hydroxyphenyl)-γ-valerolactone-3′-sulfate **8** (see Fig. 2) along with lower concentrations of other valerolactones and phenylvaleric acids such as 4*R/S*-hydroxy-5-(3′-hydroxyphenyl)valeric acid-4′-glucuronide **9**.

Using HPLC-MS-MS in conjunction with an on-line radioactivity detector [96] enabled ^14^C-SREMs and 5C-RFMs, along with ^14^C-labelled hippuric acids and low molecular weight phenolic acid catabolites, to be monitored in 0–48 h urine and feces. Urinary metabolites consisted of SREMs (20%), 5C-RFCs (42%), two and three carbon side chain-RFCs (7%) and hippuric acid (13%) together with 3′-hydroxyhippuric acid **43** (8%) along with a number of minor, unidentified radiolabelled products [51,52]. Hence, 70% of the ingested radiolabelled (−)-epicatechin was converted to microbiota-derived catabolites. The radioactivity in 0–48 h feces was associated predominantly with 4*R/S*-hydroxy-5-(phenyl)valeric acids and a smaller amount of 3-(3′-hydroxyphenyl)propanoic acid **34**.

**Fig. 8:**
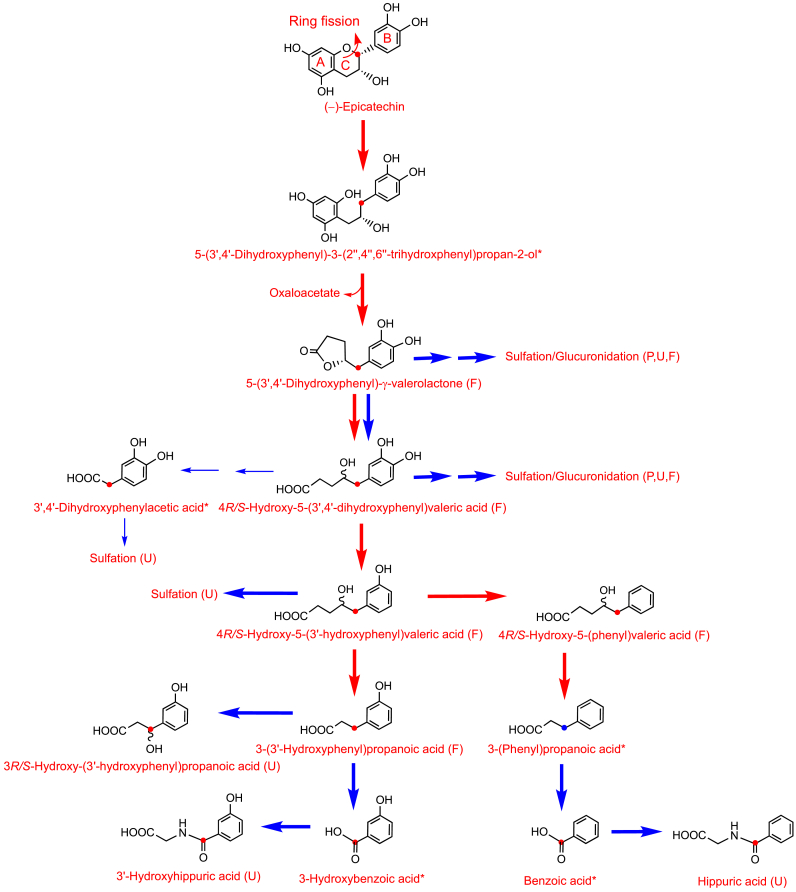
Proposed routes for the fate of [2–^14^C](−)-epicatechin passing from the small to the large intestine. Red arrows indicate microbiota-mediated conversions, and blue arrows steps catalysed by mammalian enzymes in colonocytes and/or hepatocytes. Asterisks indicate proposed intermediates that do not accumulate in detectable quantities. Detected in plasma (P), urine (U) and feces (F). The red circles indicates the position of ^14^C-label. Fine arrows are potential minor routes. Based on the data of Ottaviani et al. [51] and Borges et al. [52]. (For interpretation of the references to color in this figure legend, the reader is referred to the Web version of this article.)

Proposed pathways, via which [2–^14^C]EC entering the colon is subject to a microbiota-mediated catabolism, a well as step involving human enzymes, that lead to benzoic acids which are subjected to hepatic glycination and converted to hippuric acids, are summarised in Fig. 8. The high recovery of radioactivity originating from the ingested [2–^14^C]EC demonstrates that benzoic acids are metabolized primarily to hippuric acids, with very limited, if any conversion to C_6_–C_0_ benzene derivatives by the colonic microbiota, as this would have result in the loss of the^14^C label with removal of the carboxyl group.

**Fig. 9:**
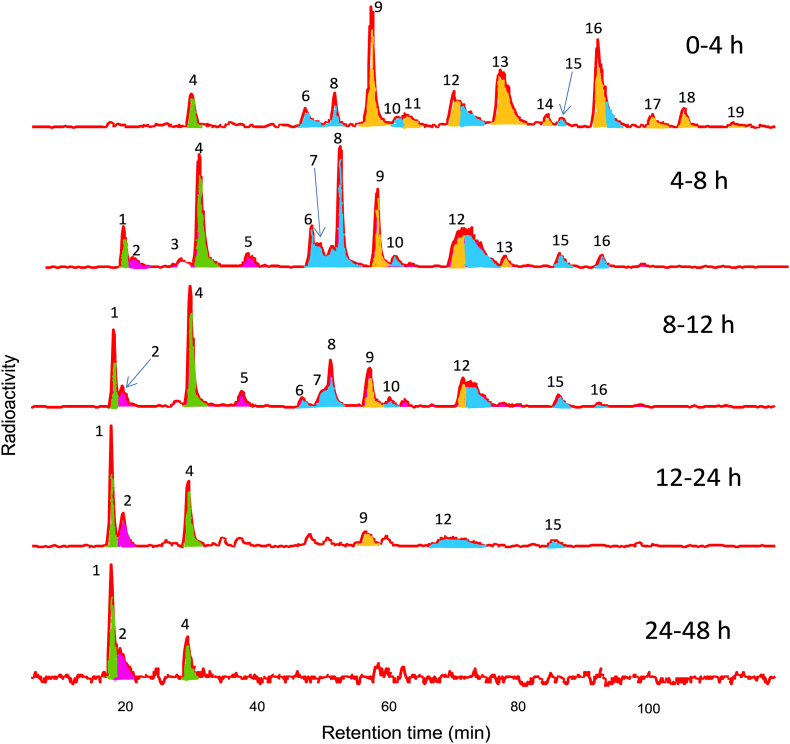
Gradient elution reversed phase HPLC with an on-line radioactivity monitor. Analysis of urine collected 0–4, 4–8, 8–12, 12–24 and 24–48 h after the ingestion of [2–^14^C](−)-epicatechin by one subject. Peak identifications: (**1**) 3′-hydroxyhippuric acid, (**2**) 3-(3′,4′-dihydroxyphenyl)propionic acid, (**3**) unknown, **4**) hippuric acid, **5**) a hydroxyphenylacetic acid-sulfate, **6**) a 4*R/S*-5-(phenyl)-γ-valerolactone-sulfo-glucuronide, (**7**) 4*R/S*-hydroxy-5-(3′-hydroxyphenyl)valeric acid-4′-sulfate, (**8**) 4*R/S*-hydroxy-5-(4′-hydroxyphenyl)valeric acid-3′-sulfate, (**9**) (−)-epictechin-3′-glucuronide, (**10**) a 4*R/S*-hydroxy-5-(phenyl)valeric acid-sulfate, (**11**) (−)-epictechin-5-sulfate, (**12**) an (−)-epicatechin-sulfo-glucuronide, and 4*R/S*-5-(4′-hydroxyphenyl)-γ-valerolactone-3′-sulfate, (**13**) (−)-epictechin-3′-sulfate, (**14**) 3′-methoxy-(−)-epicatechin-4′-sulfate, (**15**) unknown, (**16**) 3′-methoxy-(−)-epicatechin-5-sulfate and 4*R/S*-5-(3′-methoxyphenyl)-γ-valerolactone-4′-sulfate, (**17**–**19**) methoxy-(−)-epicatechin-sulfates. Peak colors: orange – structurally-related epicatechin metabolites, blue – C_6_–C_5_ catabolites, red – C_6_–C_2/3_ catabolites, green – hippuric acids. Based on data of Ottaviani et al. [51] and Borges et al. [52]. (For interpretation of the references to color in this figure legend, the reader is referred to the Web version of this article.)

The use of [2–^14^C]EC had a specific activity of 14.5 μCi μmol^−1^ was invaluable in monitoring the overall levels of radioactivity in body fluids after supplementation, and in following the diversity of metabolites and catabolites in urine as shown in the HPLC radioactivity traces illustrated in Fig. 9. However, it was not possible to distinguish the ^14^C molecular ion from the much bigger ^12^C ion by MS because of the low intensity signal of the ^14^C^+2^ ion. This was due to i) only one of the fifteen EC carbons being labelled with ^14^C and ii) with a specific activity of 14.5 μCi μmol^−1^, the C-2 position contained both ^12^C- and ^14^C molecules with ^12^C predominating, comprising >90% of the mixture. As a consequence MS identifications of metabolites in radioactive HPLC peaks were based on co-chromatography with unlabelled ^12^C standards with the level of radioactivity in peaks facilitating accurate quantification. A further limitation is that because of the low intermittent rate of decay of the ^14^C label, HPLC radioactivity detectors require at least a *ca.* 10 s time constant to achieve useable signal/noise ratios [98]. This results in peak tailing which impacts adversely on chromatographic resolution, necessitating the use of a long shallow HPLC mobile phase gradient, such as that used in Fig. 9. Of necessity this precludes the use of rapid UHPLC. However, employing extensively labelled ^13^C instead of ^14^C-substrates, enables ^13^C ions to be readily distinguished from ^12^C fragments by MS, sacrificing the need for a detector time constant, and thus facilitating the use of rapid analysis UHPLC.

### 7.2 [6,8,10,3′,5′-^13^C_5_]Cyanidin-3-O-glucoside

**Fig. 10:**
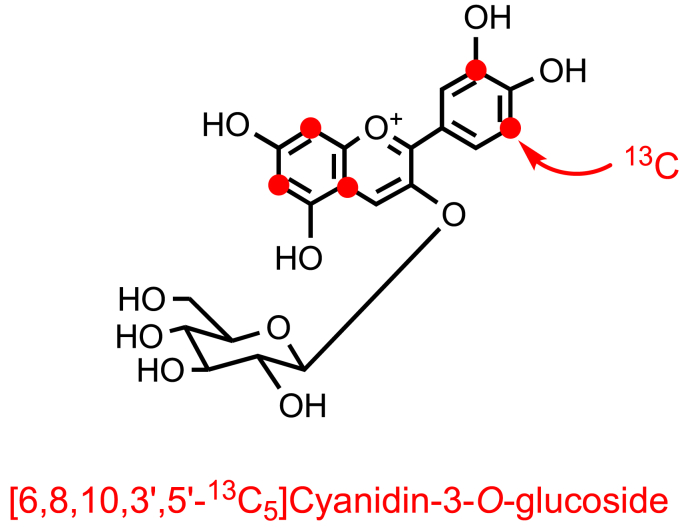
Structure and ^13^C labelling of [6,8,10,3′,5′-^13^C_5_ ]cyanidin-3-*O*-glucoside [99]. The red circles indicates the positions of ^13^C-label. (For interpretation of the references to color in this figure legend, the reader is referred to the Web version of this article.)

A^13^C-labelled anthocyanin, namely [6,8,10,3′,5′-^13^C_5_]cyanidin-3-*O*-glucoside, was synthesized by Zhang et al. [99]. The anthocyanin had a 99% ^13^C enrichment at each of the labelled positions, and as three ^13^C molecules were incorporated into the A-ring and two into the B-ring (Fig. 10) it was possible with MS detection to ascertain whether catabolites were derived from the A- or B-ring of cyanidin.

In a bioavailability study reported by Czank et al. [74] and de Ferrars et al. [97], after an overnight fast, 8 participants each ingested 0.5 g (1114 μmol) of the ^13^C_5_-anthocyanin. Blood was collected 0, 0.5, 1, 2, 4 6, 24 and 48 h after intake and urine and feces after 0–6, 6–24 and 24–48 h. Labelled CO_2_ was measured in breath collected after 0, 1, 2,4, 6, 24 and 48 h. Measuring overall levels of recovered ^13^C was not as straight forward as monitoring ^14^C with liquid scintillating counting [51,52], and necessitated the use of isotopic ratio-mass spectrometry as described by Morrison et al. [[100], [101]]. This showed mean recoveries of ^13^C to be 5.4 ± 0.7% in urine, 6.9 ± 1.6% in breath and 32 ± 6% in feces which gives a relative bioavailability of ≥12.3 ± 1.4% calculated from the combined elimination in urine and breath. The metabolite profile of the ^13^C-anthocyanin is, therefore, very different to that of [2–^14^C]EC where there was an 82% recovery of the ^14^C-label in urine and 12% in feces (51,52).

An array of ^13^C-labelled catabolites was detected in serum, urine and feces after intake of the [^13^C]cyanidin-3-*O*-glucoside. These are summarised in Table 3, Table 4 where Kay et al. nomenclature [40] replaces synonyms used by de Ferrars et al. [97]. In addition, estimates of compounds in urine and feces that were originally given in μg have been converted to μmol values. All but three of the >20 catabolites detected were ^13^C_2_ rather than ^13^C_3_-labelled compounds indicating that they originated from the B-ring of cyanidin-3-*O*-glucoside.

**Table 3:** Recovery of^13^C-labelled anthocyanins and phenolic catabolites in plasma and urine collected 0–48 h after the ingestion of 1114 μmol^13^C_5_-cyanidin-3-*O*-glucoside by 8 subjects. Figures in italicized parentheses indicate the number of volunteers in which individual compounds were detected. Based on the data of de Ferrars et al. [97].

^13^C-Labelled anthocyanins and phenolic catabolites	Plasma		Urine	
	*T*_max_ (h)	*C*_max_ (nmol/L)	Peak excretion (h)	Total 0–48 h recovery (μmol)
^*13*^*C*_*5*_*-Anthocyanins*				
Cyanidin-3-*O*-glucoside	1.8	141 ± 70 *(5)*	1–2	0.24 ± 0.11 *(7)*
Cyanidin-glucuronide^a^	–	–	1–2	0.07 ± 0.03 (5)
Peonidin-3-*O*-glucoside	–	–	1–2	0.05 ± 0.02 *(7)*
Methoxycyanidin-glucuronide^b^	–	–	1–2	0.004 ± 0.002 *(4)*
Methoxycyanidin-3-*O*-glucoside-glucuronide	–	–	1–2	0.11 ± 0.05 *(5)*
*Total anthocyanins*				*0.47*

^*13*^*C*_*2*_*-cinnamic and phenylacetic acids*				
4′-Hydroxy-3′-methoxycinnamic acid	8.2	827 ± 371 *(7)*	24–48	4.1 ± 1.5 *(8)*
4′-Hydroxyphenylacetic acid	–	–	4–5	0.33 ± 0.13 *(3)*
3′,4′-Dihydroxyphenylacetic acid	–	–	24–48	0.18 *(1)*
*Total*				*4.6*

^*13*^*C*_*2*_*-benzoic acids and benzaldehydes*				
3,4-Dihydroxybenzoic acid	3.3	146 ± 74 *(8)*	1–2	0.46 ± 0.13 *(8)*
3/4-Hydroxybenzoic acids	–	–	1–2	0.09 ± 0.03 *(5)*
4-Hydroxy-3-methoxybenzoic acid	12.5	1845 ± 838 *(2)*	1–2	5.6 ± 2.1 *(4)*
3-Hydroxy-4-methoxybenzoic acid	2.0	195 *(1)*	1–2	0.47 ± 0.32 *(4)*
3,4-Dihydroxy-methoxybenzoic acid	8.4	12 ± 5 *(8)*	3–4	0.11 ± 0.04 *(8)*
Benzoic acid-4-glucuronide	10.9	74 ± 20 *(7)*	4–5	0.17 ± 0.06 *(7)*
3-Hydroxybenzoic acid-4-glucuronide	3.8	68 ± 61 *(8)*	1–2	0.30 ± 0.12 *(8)*
4-Hydroxybenzoic acid-3-glucuronide	2.7	11 ± 3 *(5)*	1–2	1.8 ± 0.1 *(8)*
3-Hydroxybenzoic acid-4-sulfate	11.4	157 ± 116 *(8)*	1–2	2.1 ± 0.8 *(8)*
4-Hydroxybenzoic acid-3-sulfate			1–2	1.4 ± 0.4 *(8)*
3-Methoxybenzoic acid-4-glucuronide	4.8	24 ± 4 *(8)*	4–5	1.8 ± 0.3 *(8)*
3-Methoxybenzoic acid-4-sulfate	30	430 ± 299 *(4)*	3–4	1.8 ± 0.4 *(7)*
4-Methoxybenzoic acid-3-glucuronide	4.3	35 ± 5 *(8)*	5–6	1.5 ± 0.3 *(8)*
4-Methoxybenzoic acid-3-sulfate	–	–	3–4	0.74 ± 0.37 *(5)*
4-Hydroxybenzaldehyde	5.6	667 ± 653 *(7)*	5–6	0.08 ± 0.07 *(2)*
3,4-Dihydroxybenzaldehyde	–	–	0–1	0.05 ± 0.02 *(6)*
*Total benzoic acid and benzaldehydes*				*18.5*

^*13*^*C*_*2*_*-hippuric acid*				
Hippuric acid	15.7	1962 ± 1389 *(8)*	6–24	13.3 ± 1.2 *(8)*
*Total*				*13.3 (1.2% recovery)*

^*13*^*C*_*3*_*-Catabolites*				
4′-Hydroxy-3′-methoxycinnamic acid	13.3	87 ± 38 *(6)*	0–1	1.1 ± 0.9 *(8)*
2-Hydroxy-4-methoxybenzoic acid	–	–	3–4	0.38 *(1)*
2,4,6-Trihydroxybenzaldehyde	2.8	582 ± 536 *(4)*	6–24	0.42 ± 0.13 *(8)*
*Total*	–	–	–	*1.5*
*Total recovery of anthocyanins and phenolic catabolites*	*–*	*–*	*–*	*38.*4 μmol*, 3.4% of intake*

a Two isomers.

b Three isomers, – not detect/not estimated.

**Table 4:** Recovery of^13^C-labelled anthocyanins and phenolic catabolites in feces collected 0–48 h after the ingestion of 1114 μmol^13^C_5_-cyanidin-3-*O*-glucoside by 8 subjects. Figures in italicized parentheses indicate the number of volunteers in which individual compounds were detected. Based on data of de Ferrars et al. [97].

^13^C-Labelled anthocyanins and phenolic catabolites	Total recovery (μmol)
^*13*^*C*_*5*_*-Anthocyanins*	
Cyanidin-3-*O*-glucoside	0.82 *(1)*
*Total*	*0.82*
^*13*^*C*_*2*_*-cinnamic and phenylacetic acids*	
3′,4′,-Dihydroxycinnamic acid	2.1 *(2)*
4′-Hydroxy-3′-methoxycinnamic acid	19.5 *(6)*
3′,4′-Dihydroxyphenylacetic acid	0.14 *(5)*
4′-Hydroxyphenylacetic acid	0.41 *(1)*
*Total*	*22.2*
^*13*^*C*_*2*_*-Benzoic acids and benzaldehydes*	
3,4-Dihydroxybenzoic acid	3.7 *(7)*
2,3-Dihydroxybenzoic acid	2.8 *(2)*
Hydroxybenzoic acid	0.12 *(3)*
2-Hydroxy-4-methoxybenzoic acid	1.6 *(2)*
4-Hydroxy-3-methoxybenzoic acid	0.61 *(3)*
3-Hydroxy-4-methoxybenzoic acid	0.12 *(1)*
3,4-Dihydroxy-methoxybenzoic acid	1.4 *(6)*
Dimethoxybenzoic acid	0.19 *(1)*
Benzoic acid-4-glucuronide	0.07 *(8)*
3-Hydroxybenzoic acid-4-glucuronide	0.11 *(1)*
4-Hydroxybenzoic acid-3-glucuronide	0.22 *(3)*
3-Hydroxybenzoic acid-4-sulfate	0.11 *(3)*
4-Hydroxybenzoic acid-3-sulfate	0.18 *(4)*
3-Methoxybenzoic acid-4-glucuronide	0.01 *(3)*
3-Methoxybenzoic acid-4-sulfate	0.04 *(3)*
4-Methoxybenzoic acid-3-glucuronide	0.04 *(3)*
4-Methoxybenzoic acid-3-sulfate	0.71 *(2)*
3,4-Dihydroxybenzaldehyde	0.06 *(6)*
4-Hydroxybenzaldehyde	0.01 *(2)*
4-Methoxybenzaldehyde	0.15 *(1)*
*Total*	*12.2*
^*13*^*C*_*2*_*-Hippuric acid*	
Hippuric acid	0.22 *(3)*
*Total*	*0.22*
^*13*^*C*_*3*_*-Catabolites*	
4′-Hydroxy-3′-methoxycinnamic acid	3.6 *(5)*
2,4,6-Trihydroxybenzaldehyde	0.70 *(8)*
*Total*	*4.3*
*Total recovery of anthocyanins and phenolic catabolites*	*39.*7 μmol*, 3.5% of intake*

While there were some similarities, many more urinary phenolics were detected in the [^13^C_5_]cyanidin-3-*O*-glucoside study than in the raspberry cyanidin-3-*O*-sophoroside feed by Ludwig et al. [60]. However, a number of ^13^C-catabolites were detected in relatively small quantities in samples from ≤4 of the 8 subjects who participated in the study (Table 3, Table 4). Furthermore, there were much more marked volunteer-to-volunteer variations in the ^13^C-labelled study than in the study in which raspberries were ingested [60]. There were also quantitative and qualitative differences in the catabolic profiles, such as the more complex excretion of ^13^C_2_-benzoic acid conjugates. These differences could be a consequence of the ingestion of the 1114 μmol bolus of [^13^C_5_]cyanidin-3-*O*-glucoside compared with the 292 μmol of anthocyanins that were ingested in a raspberry matrix, Arguably, the high ^13^C dose exaggerated both the inherent instability of the cyanidin moiety [102] and volunteer differences in the capacity of their colonic microbiota to catabolise the anthocyanin.

### 7.3 Excretion of ^14^C-hippuric acid derived from [^14^C]-(−)-epicatechin and background [^12^C]-hippuric acid

The 0–12 h urinary excretion of unlabelled ^12^C-hippuric acid **26** by subjects with a full GI tract over a 12 h fasting period, after prior adherence to a low (poly)phenol diet for 36 h, was 366 ± 28 μmol (Table 2) [39]. Excretion of [^14^C]hippuric acid over a similar period, after ingestion of 207 μmol [^14^C](−)-epicatechin, was 13.4 ± 2.9 μmol [52] which corresponds to 7.2% of intake. The size of the endogenous hippuric acid pool was not determined in the ^14^C-investigation. However, using the 366 ± 28 μmol in Table 2 as a guide, indicates that the combined [^12^C]- and [^14^C]-hippuric acid pool was 379 μmol of which the radiolabelled flavan-3-ol metabolite was a minor contributor at 3.5% (Table 5). The vast majority of the hippuric acid, and presumably many of the related phenolic acids, therefore, appear to originate predominantly from other sources with the available evidence pointing principally to phenylalanine and tyrosine. This would also appear to be the case in the [^13^C_5_]cyanidin-3-*O*-glucoside study [74,97] where analysis of 0–48 h urine indicated a 1.2% conversion to [^13^C_2_]hippuric acid (Table 5).

**Table 5:** Twelve-hour urinary excretion of^12^C-hippuric acid by subjects on a low (poly)phenol diet [12] compared with excretion of^14^C-hippuric acid following the ingestion of 207 μmol [^14^C](−)-epicatechin [51,52]. Subjects in both groups have a full gastrointestinal tract.

Source	0–12 h urinary excretion
Baseline with colon	^12^C-hippuric acid: 366 ± 28 μmol
207 μmol [^14^C]-(−)-Epicatechin	^14^C-hippuric acid:13.4 ± 2.9 μmol (7.2% of intake) Total^12/14^C-hippuric acid 379 μmol (^14^C - 3.5%)

## 8 Summary

There are number of methods for obtaining information to help distinguish between i) phenolics derived from microbiota-mediated breakdown of dietary (poly)phenols, and ii) the large quantities of phenolics originating from other sources which involve the aromatic amino acids phenylalanine **1** and tyrosine **2**, and to a lesser extent catecholamines. Feeding studies using isotopically-labelled substrates remain the gold standard but for a several reasons, principally cost and the difficulties in obtaining ethical permission, they have been used only rarely. Investigations involving the ingestion of plant-derived products containing (poly)phenols, such as ellagitannins, flavan-3-ols, isoflavones, and lignans, have the advantage of being converted by microbiota to phenolic catabolites that have never been reported as products of amino acid metabolism. Nevertheless, investigations involving other (poly)phenols, and designed to at least partially disentangle the production of phenolic catabolites from amino acids and dietary (poly)phenols, are to be encouraged.

Whatever *in vitro* or *in vivo* test system is used, UPLC-HR-MS and UHPLC-QQQ-MS are now the analytical methods of choice. Accurate qualitative and quantitative estimates require the use of standards of phenolics and their phase II metabolites. Although there are methods for the synthesis of relevant compounds [103,104], and many are becoming available from commercial sources, because of their number it is all but impossible for investigators to obtain a full complement of the standards that are required. In the circumstances, information should use validated analytical methodology [43,[105], [106], [107]], and the basis of identifications and quantifications should be categorized according to the Metabolic Standards Initiative Metabolite Identification levels of Sumner et al. [108].

Finally, it is becoming apparent that phenylalanine and tyrosine, originating from both turnover of endogenous proteins and dietary sources, can make a substantial contribution to the pools of low molecular weight phenolic catabolites. The potential impact on health of catabolites derived from the various sources of the aromatic amino acids requires further detailed investigation, including updated information on their metabolism and bioactivity. However, any protective effects from this source will be supplemented by phenolic catabolites generated from regular intake of plant-based foods and beverages that are rich in a diversity of (poly)phenols as well as phenylalanine and tyrosine. Clarifying the contributions to the pools of circulating bioactive phenolic catabolites is essential for future, informed dietary health recommendations.

## Contributions

M.N.C and A.C. drafted the text, with assistance from P.M. and I.A.L., and monitored edits from the other authors who also provided additional data, helped prepare figures and approved the final text.

## Funding

A.C., G.P.-C., G. I.R.G, and T.M.A. were funded by the 10.13039/501100002383Distinguished Scientist Fellowship Program (DSFP) of King Saud University, Riyadh, Saudi Arabia. I.A.L. was supported by the 10.13039/501100017266Gobierno de Navarra: Grant no. 0011-3947-2021-000034. P.M. received funding from the 10.13039/501100007601European Union's Horizon 2020 Research and Innovation Programme: Grant no. 950050; and the National Recovery and Resilience Plan of the Italian Ministry of University and Research and the European Union: Project code PE00000003.

## CRediT authorship contribution statement

**Michael N. Clifford:** Conceptualization, Writing – original draft. **Iziar A. Ludwig:** Writing – review & editing. **Gema Pereira-Caro:** Formal analysis, Writing – review & editing. **Laila Zeraik:** Writing – review & editing. **Gina Borges:** Conceptualization, Writing – review & editing. **Tahani M. Almutairi:** Conceptualization. **Sara Dobani:** Writing – review & editing. **Letizia Bresciani:** Writing – review & editing. **Pedro Mena:** Conceptualization, Writing – review & editing. **Chris I.R. Gill:** Writing – review & editing. **Alan Crozier:** Conceptualization, Writing – original draft, Writing – review & editing.

## Declaration of competing interest

The authors declare that they have no known competing financial interests or personal relationships that could have appeared to influence the work reported in this paper.None

## Data Availability

Data will be made available on request.
