# Supplementary material for: Exploring and disentangling the production of potentially bioactive phenolic catabolites from dietary (poly)phenols, phenylalanine, tyrosine and catecholamines
Source: Redox Biol. 2024 Feb 13;71:103068. doi: 10.1016/j.redox.2024.103068 (PMC10891336; doi:10.1016/j.redox.2024.103068)
Supplement: Supplementary file 1 [file mmc1.docx]

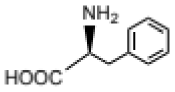

L-Phenylalanine **1**

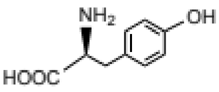

L-Tyrosine **2**

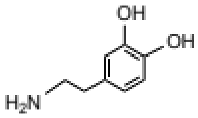

Dopamine **3**

Structures 1-3

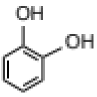

1,2-Dihydroxybenzene **4**

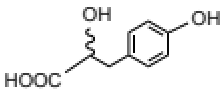

2*R*/*S*-Hydroxy-3-(4'-hydroxyphenyl)propanoic acid **5**

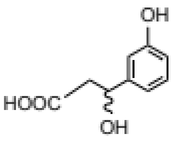

3*R*/*S*-Hydroxy-3-(3'-hydroxyphenyl)propanoic acid **6**

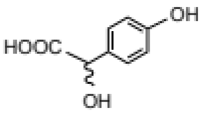

2*R*/*S*-Hydroxy-(4'-hydroxyphenyl)acetic acid **7**

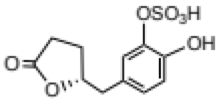

5-(4'-Hydroxyphenyl)-γ-valerolactone-3'-sulfate **8**

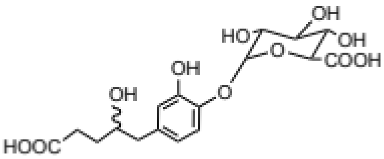

4*R*/*S*-Hydroxy-5-(3'-hydroxyphenyl)valeric acid-4'-glucuronide **9**

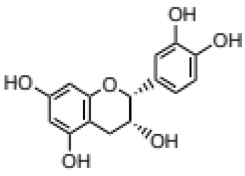

(–)-Epicatechin **10**

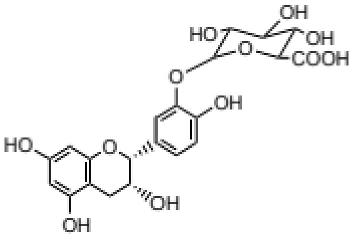

(–)-Epicatechin-3'-glucuronide **11**

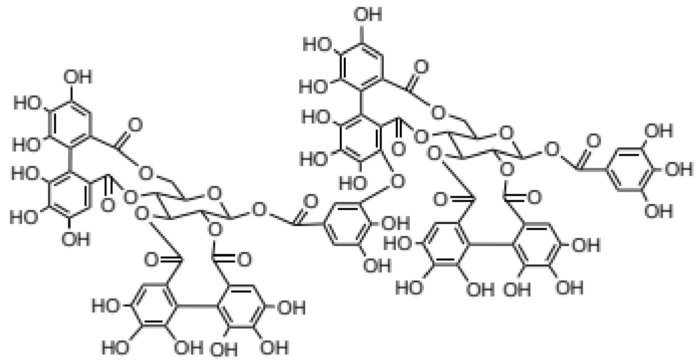

Sanguiin H-6 **12**

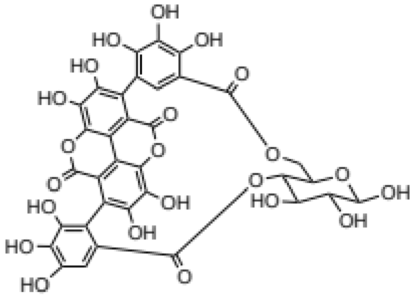

Punicalin **13**

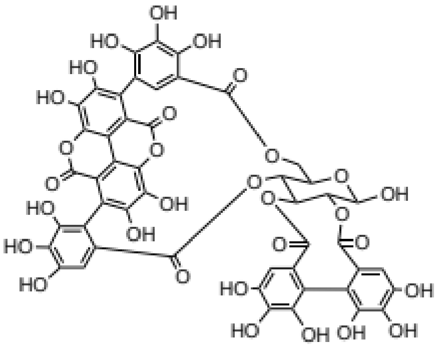

Punicalagin **14**

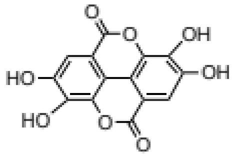

Ellagic acid **15**

Structures 12-15

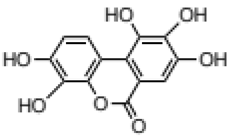

3,4,8,9,10-Pentahydroxy-urolithin **16**
(aka urolithin M5)

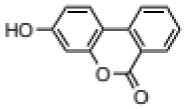

3-Hydroxy-urolithin **17**
(aka urolithin B)

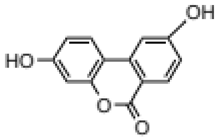

3,9-Dihydroxy-urolithin **18**
(aka iosurolithin A)

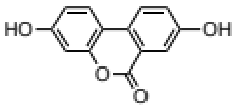

3,8-Dihydroxy-urolithin **19**
(aka urolithin A)

Structures 16-19

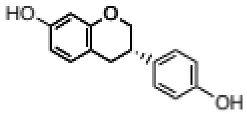


*S*-Equol **20**

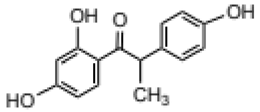


*O*-Desmethylangolensin **21**

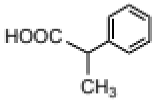


2-(Phenyl)propanoic acid **22**

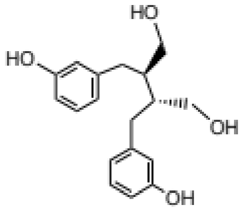


Enterodiol **23**

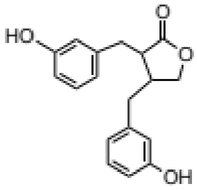


Enterolactone **24**

Structures 20-24

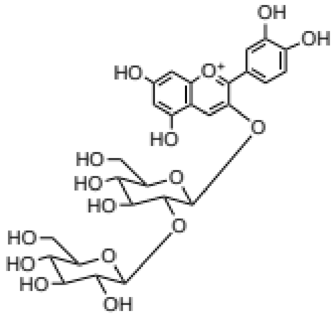


Cyanidin-3-*O*-sophoroside **25**

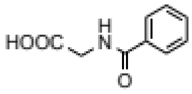


Hippuric acid **26**

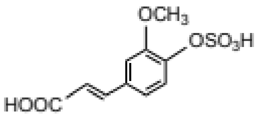


3'-Methoxycinnamic acid-4'-sulfate **27**

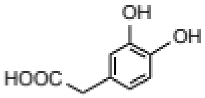


3',4'-Dihydroxyphenylacetic acid 2**8**

Structutes 25-28

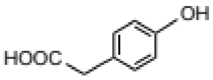

4'-Hydroxyphenylacetic acid 29

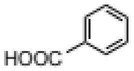

Benzoic acid **30**

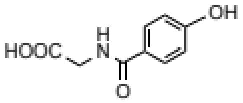

4'-Hydroxhippuric acid **31**

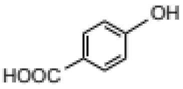

4-Hydroxybenzoic acid **32**

Structures 29-32

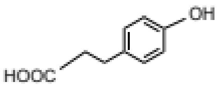

3-(4'-Hydroxyphenyl)propanoic acid **33**

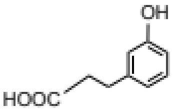

3-(3'-Hydroxyphenyl)propanoic acid **34**

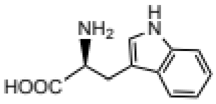

Tryptophan **35**

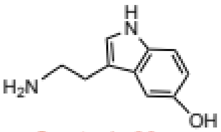

Serotonin **36**

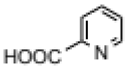

Picolinic acid **37**

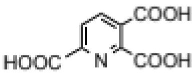

Quinolinic acid **38**

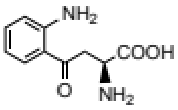

Kynurenine **39**

Structures 33 - 39

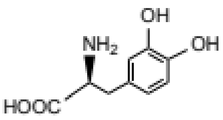

3',4'-Dihydroxyphenylalanine **40**
(aka L-DOPA)

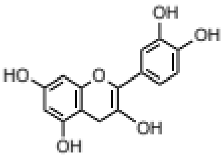

Quercetin **41**

Structures 40-41

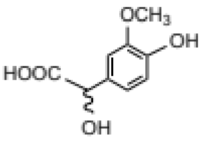

2*R*/*S*-Hydroxy-(4'-hydroxy-3'-hydroxyphenyl)acetic acid **42**

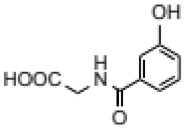

3'-Hydroxyhippuric acid **43**

Structures 42-43

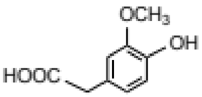

4'-Hydroxy-3'-methoxyphenylacetic acid **44**

Structure 44

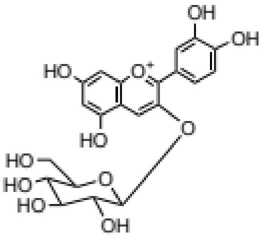

Cyanidin-3-*O*-glucoside **45**

Structure 45
